# MRI features of the normal prostatic peripheral zone: the relationship between age and signal heterogeneity on T2WI, DWI, and DCE sequences

**DOI:** 10.1007/s00330-020-07545-7

**Published:** 2021-01-04

**Authors:** Vlad Bura, Iztok Caglic, Ziga Snoj, Nikita Sushentsev, Alexandra S. Berghe, Andrew N. Priest, Tristan Barrett

**Affiliations:** 1 Department of Radiology, County Clinical Emergency Hospital, Cluj-Napoca, Cluj Romania; 2 Department of Radiology, Addenbrooke’s Hospital and University of Cambridge, Box 218, Hills Road, Cambridge, CB2 0QQ UK; 3 Radiology Institute, University Medical Centre Ljubljana, Ljubljana, Slovenia; 4 Department of Medical Informatics and Biostatistics, Iuliu Hatieganu University of Medicine and Pharmacy, Cluj-Napoca, Romania

**Keywords:** Multiparametric MRI, Prostate cancer, Normal anatomy, Anatomy, Aging

## Abstract

**Objectives:**

To assess the multiparametric MRI (mpMRI) appearances of normal peripheral zone (PZ) across age groups in a biopsy-naïve population, where prostate cancer (PCa) was subsequently excluded, and propose a scoring system for background PZ changes.

**Methods:**

This retrospective study included 175 consecutive biopsy-naïve patients (40–74 years) referred with a suspicion of PCa, but with subsequent negative investigations. Patients were grouped by age into categories ≤ 54, 55–59, 60–64, and ≥ 65 years. MpMRI sequences (T2-weighted imaging [T2WI], diffusion-weighted imaging [DWI]/apparent diffusion coefficient [ADC], and dynamic contrast-enhanced imaging [DCE]) were independently evaluated by two uro-radiologists on a proposed 4-point grading scale for background change on each sequence, wherein score 1 mirrored PIRADS-1 change and score 4 represented diffuse background change. Peripheral zone T2WI signal intensity and ADC values were also analyzed for trends relating to age.

**Results:**

There was a negative correlation between age and assigned background PZ scores for each mpMRI sequence: T2WI: *r* = − 0.52, DWI: *r* = − 0.49, DCE: *r* = − 0.45, *p* < 0.001. Patients aged ≤ 54 years had mean scores of 3.0 (T2WI), 2.7 (DWI), and 3.1 (DCE), whilst patients ≥ 65 years had significantly lower mean scores of 1.7, 1.4, and 1.9, respectively. There was moderate inter-reader agreement for all scores (range *κ* = 0.43–0.58). Statistically significant positive correlations were found for age versus normalized T2WI signal intensity (*r* = 0.2, *p* = 0.009) and age versus ADC values (*r* = 0.33, *p* = 0.001).

**Conclusion:**

The normal PZ in younger patients (≤ 54 years) demonstrates significantly lower T2WI signal intensity, lower ADC values, and diffuse enhancement on DCE, which may hinder diagnostic interpretation in these patients. The proposed standardized PZ background scoring system may help convey the potential for diagnostic uncertainty to clinicians.

**Key Points:**

• *Significant, positive correlations were found between increasing age and higher normalized T2-weighted signal intensity and mean ADC values of the prostatic peripheral zone.*

• *Younger men exhibit lower T2-weighted imaging signal intensity, lower ADC values, and diffuse enhancement on dynamic contrast-enhanced imaging, which may hinder MRI interpretation.*

• *A scoring system is proposed which aims towards a standardized assessment of the normal background PZ. This may help convey the potential for diagnostic uncertainty to clinicians.*

**Supplementary Information:**

The online version contains supplementary material available at 10.1007/s00330-020-07545-7.

## Introduction

Prostate cancer (PCa) is the second commonest male cancer and the fifth leading cause of cancer-related deaths in men worldwide [1]. The use of the prostate-specific antigen (PSA) test for screening symptomatic patients in the mid-1990s dramatically changed the profile of PCa patients, and within 10 years of its introduction, the average age at presentation was significantly lowered [2]. The US Preventive Services Task Force recently recommended that PSA screening can be selectively offered to men aged 55 to 69 years [3]; it is therefore likely that trends towards a younger age at presentation will continue. International guidelines now recommend multiparametric (mp) MRI of the prostate as the initial diagnostic test in men presenting with a suspicion of PCa [4, 5], making knowledge of common pitfalls, normal prostatic appearance, and anatomical variants essential for accurate lesion detection at MRI [6–8].

The Prostate Imaging Reporting and Data System (PIRADS) guidelines were initially developed by the European Society of Urogenital Radiology in 2012 and subsequently updated in 2015 and 2019, with the aim of standardizing the way prostate MRI is performed, interpreted, and reported [9–11]. MRI interpretation is based on a 5-point scale, where score 1 describes the normal MRI appearances of the prostate and score 5 indicates that clinically significant PCa is highly likely. The majority of prostate tumors arise from the peripheral zone (PZ) [12]. On T2-weighted imaging (T2WI), PIRADS category 1 change describes the normal PZ as being of uniform hyperintensity. PIRADS category 2–3 change in the PZ includes wedge-shaped to diffuse intermediate-to-low T2WI background signal intensity changes which are non-specific and typically benign. A variety of conditions can result in these T2WI appearances, including post-biopsy hemorrhage, prostatitis, atrophy, fibrosis, and post-treatment change [13, 14]. Due to the limited specificity of these features, PIRADS assigns diffusion-weighted imaging (DWI) as the dominant sequence and dynamic contrast-enhanced imaging (DCE) as the secondary sequence for assessment of the PZ. However, T2WI is essential for local staging of PCa and may have a role to play in the context of unenhanced biparametric MRI, or when artifact affects the quality of the DWI and/or DCE imaging [15].

The prostate is known to increase in volume with age [16, 17], and there is some limited evidence to suggest that diffuse low T2WI change in the PZ can represent a normal anatomical feature in younger patients [18, 19]. Such change may also result in mild restricted diffusion and diffuse early enhancement on DCE sequences, which may therefore hinder mpMRI interpretation and potentially mask clinically significant PCa. A systematic assessment of the background gland is not currently included within the PIRADS guidelines; however, such a tool is routine for breast imaging [20] and may also prove beneficial for prostate MRI interpretation. Mapping the normal age-related appearance of the PZ is of further relevance given the trend towards a younger age of patients presenting with suspected PCa. Thus, the purpose of this study was to assess the MRI appearance of the PZ across age groups in a biopsy-naïve population, where PCa was subsequently excluded, and to propose a scoring system for background PZ changes according to MRI findings.

## Materials and methods

This single-institution retrospective study was performed using a prospectively maintained database, collected as part of a service evaluation of the prostate diagnostic pathway, with the need for informed consent for data analysis waived by the Local Ethics Committee.

### Eligibility criteria and patient characteristics

The inclusion criteria for our study were as follows: (a) patients referred with a suspicion of PCa who underwent prostate mpMRI between October 2015 and January 2019 and with no history of biopsy prior to MRI; (b) either a negative MRI or diffuse (non-focal) PIRADS 3 change reported on MRI; (c) a subsequent negative systematic (US-guided, via either a transrectal or transperineal approach; minimum 12 cores) prostate biopsy or at least 1-year clinical follow-up (*n* = 14; mean 24.2 months’ follow-up, range 21–32 months) to exclude malignancy. The exclusion criteria were as follows: (a) severe artifacts on MRI (including total hip replacement or other pelvic metalwork; *n* = 4); (b) the presence of prostatic intraepithelial neoplasia (PIN) (*n* = 3), high-grade PIN (*n* = 3), or atypical small acinar proliferation (ASAP) at pathology (*n* = 1); (c) pathologically confirmed acute or chronic prostatitis (*n* = 5). The final study population included a total of 175 patients.

### MRI parameters

Patients underwent prostate MRI on either at 1.5 T (10/175 patients, Discovery MR450 or Optima MR450w, GE Healthcare) or at 3 T (165/175 patients, Discovery MR750, GE Healthcare), with a 32-channel phased array coil or a 16-channel anterior phased array coil plus built-in table coils. Axial T1-weighted fast spin echo (FSE) of the whole pelvis and T2-weighted FSE of the prostate (axial/sagittal/coronal) images were acquired. Axial T2-weighted FSE used the following imaging parameters: at 1.5 T, echo time (TE) 86 ms, field of view (FOV) 24 × 24 cm^2^, acquisition matrix 352 × 224, slice thickness 3.5 mm with 0.5 mm gap, 4–5 signal averages; at 3 T, TE 98–107 ms, FOV 22 × 22 cm^2^, acquisition matrix 320–384 × 256, slice thickness 3 mm with 0 mm gap, 3 signal averages, repetition time (TR) 3000–5000 ms, echo train length 16, receiver bandwidth ± 31.25 or ± 41.67 kHz. DWI was performed using a spin-echo echo-planar imaging pulse sequence with *b* values of 150, 550, 750, and 1000, with 1400 at 3 T, and separate high *b* value acquisition of 1400 (1.5 T) and 2000 s/mm^2^ (3 T). Parameters at 1.5 T/3 T included as follows: TE 62/70 ms; FOV 24 × 24/28 × 28 cm^2^; slice thickness 4/3 mm, highest *b* value at 1000/1400 s/mm^2^. Both 1.5 T and 3 T used TR 2800–4500 ms, acquisition matrix 128 × 128, slice gap 0 mm, receiver bandwidth ± 111.11 kHz, and parallel imaging (ASSET) factor 2. In each case, trace-weighted images were acquired by averaging 3 orthogonal diffusion directions, and apparent diffusion coefficient (ADC) maps were automatically calculated using a maximum *b* value of 1000 s/mm^2^ and with a mono-exponential fit. DCE-MRI axial 3D fast spoiled gradient echo (FSPGR) was acquired (TR/TE 4.1/1.8 ms, FOV 24 × 24 cm^2^) following bolus injection of gadobutrol (Gadovist, Bayer Healthcare) via a power injector, rate 3 mL/s (dose 0.1 mmol/kg), temporal resolution 7 s at 3 T and 10 s at 1.5 T. Detailed parameters of 1.5/3-T MRI protocols are summarized in Supplemental table 1.

### Background score assessment

**Fig. 1:**
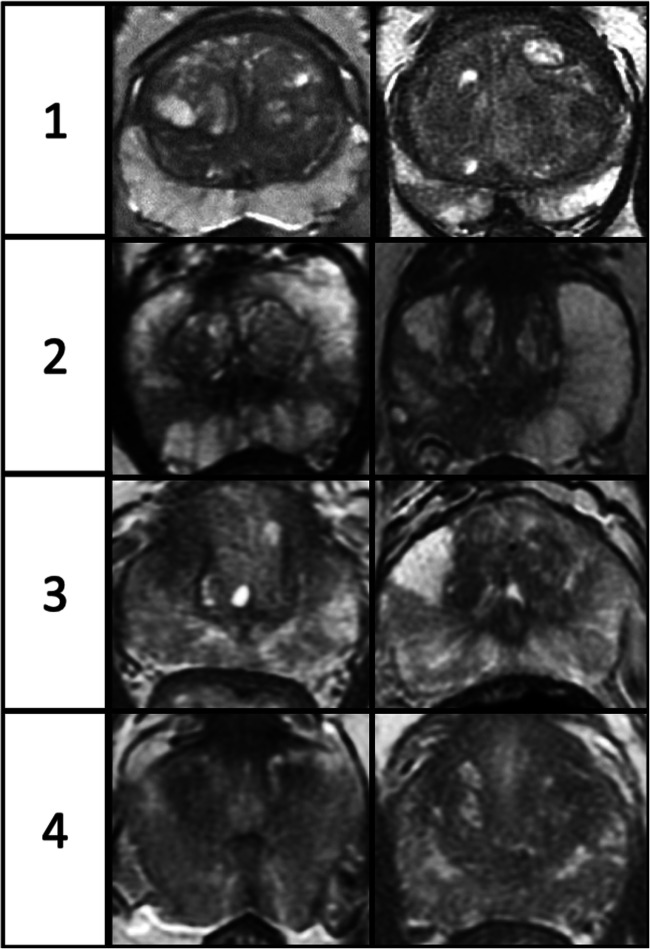
Image examples of T2-weighted imaging scores 1 to 4. Detailed descriptors can be found in Table 1

**Fig. 2:**
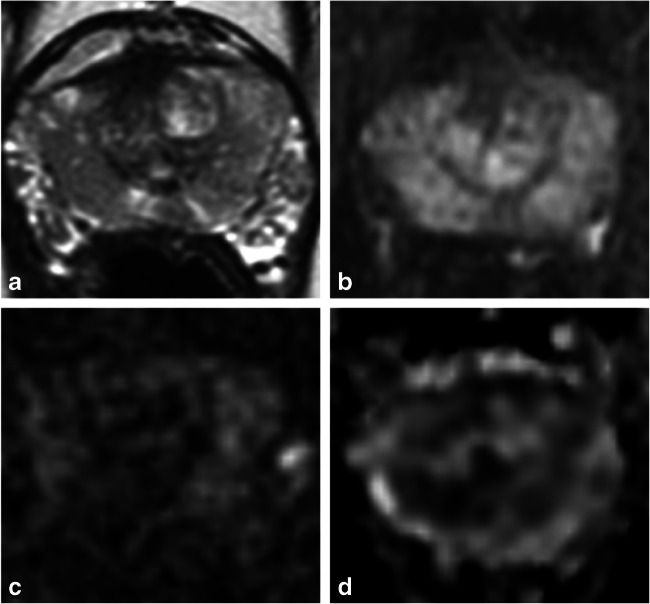
A 44-year-old man with a family history of prostate cancer, PSA 0.5 ng/mL and subsequent negative transrectal-ultrasound-guided biopsy. T2-weighted (**a**), early-phase dynamic contrast-enhanced (**b**), b-2000 diffusion-weighted (**c**), and apparent diffusion coefficient map (**d**) images show features of score 4 for each of the individual sequences

Images were independently reviewed by two uro-radiologists, blinded to patient age and clinical details, with 12 years (T.B.) and 6 years (I.C.) of experience in prostate MRI reporting and considered experts having each reported > 2000 studies [7]. MpMRI sequences were analyzed, with each evaluated on a predefined 4-point scoring scale for background change, similar to the breast composition category reported as part of the BIRADS system, used to describe breast density on mammograms [20]. Score 1 mirrors PIRADS category 1 change and score 4 represents diffuse heterogeneous change. For DWI scoring, the highest *b* value sequence and ADC maps were reviewed together. The detailed descriptors for scoring each mpMRI sequence are presented in Table 1, with examples of scores 1–4 for T2WI in Fig. 1 and an example of score 4 for T2WI, DWI, and DCE in Fig. 2.

**Table 1:** Detailed descriptors for T2WI, DWI, and DCE scoring

Score	Proportion involved (%, S*)	T2WI score description	DWI score description	DCE score description
1	< 50% 0–2 S	Almost entirely high SI	Almost entirely normal on ADC and high *b* value DWI	Predominate type 1 curve, no/minimal early enhancement
2	> 50% 1–2 S or < 50% 3–4 S	Scattered linear/wedge-shaped area/s of intermediate SI	Minor linear/wedge-shaped area/s hypointense on ADC and/or hyperintense on high *b* value DWI	Minor linear/wedge-shaped area/s of early enhancement
3	> 50% 3–4 S or ≤ 25% 5–6 S	Moderate geographical and wedge areas of intermediate SI	Moderate geographical/wedge shaped area/s hypointense on ADC and/or hyperintense on high *b* value DWI	Moderate geographical/wedge shaped area/s of early enhancement
4	Involving 5–6 S	Very heterogeneous SI with loss of zonal border or diffuse low SI	Very heterogeneous or diffuse change; may have marked change on *b* value imaging or ADC, but not both	Diffuse early enhancement

*S sector, *1 S* right or left apex/mid/base of the prostate gland

### T2WI signal intensity and ADC value assessment

Using the axial T2WI sequences of each patient, regions of interest (ROIs) were defined corresponding to the entire PZ (excluding transition zone (TZ) and central zone) and the whole gland (WG), respectively, and with equivalent ROIs for the PZ also drawn on ADC maps. Additional ROIs were drawn within the right obturator internus muscle over two consecutive MRI slices for calculation of T2WI normalization ratios. The ROIs were defined by an abdominal imaging fellow (Z.S.). The pixels from multislice ROIs were combined into volumes for analysis. The mean T2WI signal intensities and ADC values within each volume were calculated using in-house developed software developed in MATLAB (The MathWorks). T2WI signal intensity normalization ratios were obtained by dividing mean T2WI signal intensities of the PZ by those of obturator muscle in each patient. The volume measurement data for TZ (including central zone) was derived by subtracting PZ from WG volumes and respective T2WI signal intensities and ADC values within each volume.

### Statistical analysis

Patients were grouped by age into 4 a priori selected categories: (a) patients aged ≤ 54 years (*n* = 47), (b) 55–59 years (*n* = 40), (c) 60–64 years (*n* = 33), and (d) ≥ 65 years (*n* = 55). Weighted kappa (κ) scores were used to determine the agreement between the two readers in assigning scores for T2WI, DWI, and DCE, respectively. The *k* statistics were interpreted as follows: a *k* value of less than 0.20 indicated poor agreement; a *k* value of 0.21–0.40, fair agreement; a *k* value of 0.41–0.60, moderate agreement; a *k* value of 0.61–0.80, substantial agreement; and a *k* value of 0.81–1.00, almost perfect agreement [21]. The chi-square test was used to evaluate the relationship between subjective scores (T2WI and DCE) and age groups, and Fisher’s exact test was performed to assess the relationship between DWI scores and age groups. The strength of age association with the scores for the three sequences individually was appreciated through Cramér’s V coefficient, wherein a value > 0.25 is considered a strong association [22]. Spearman’s correlation was performed to assess the relationship between ordinal and non-normally distributed continuous variables; the remaining relationships were evaluated by Pearson’s correlation. A *p* value of < 0.05 was considered to be statistically significant. Statistical analysis was performed with SPSS Statistics 24.0 (IBM; SPSS, 2016).

## Results

**Fig. 3:**
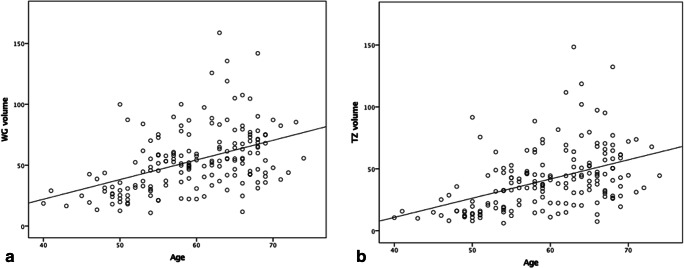
Relationships between (**a**) age and whole gland (WG) volume and (**b**) age and transition zone (TZ) volume, on MRI (*n* = 174, 1 outlier excluded). The graphs show that both WG and TZ volumes increase with increasing age

A total of 175 patients were analyzed with a mean age of 60 years (range 40–74 years), mean PSA level of 6.64 ng/mL (range 0.3–20.9 ng/mL), and mean prostate volume of 53.7 mL (range 10.9–158.8 mL) (Table 2 and Fig. 3). A moderate positive correlation was demonstrated between age and both WG volume (*r* = 0.47, *p* < 0.001) and TZ volume (*r* = 0.49, *p* < 0.001), whereas no correlation was shown for age and PZ volume (*r* = 0.07, *p* = 0.40).

**Table 2:** Age categories with age range, number of patients, and mean prostate volumes

Age range	No. of patients	Mean WG volume (mL)	Mean TZ volume (mL)	Mean PZ volume (mL)
≤ 54	47	34.9	23.8	11.1
55–59	40	54.3	41.3	13.3
60–64	33	64.5	50.4	14.1
≥ 65	55	62.5	50.1	12.4

*WG* whole gland, *TZ* transition zone, *PZ* peripheral zone

### Background scoring

**Fig. 4:**
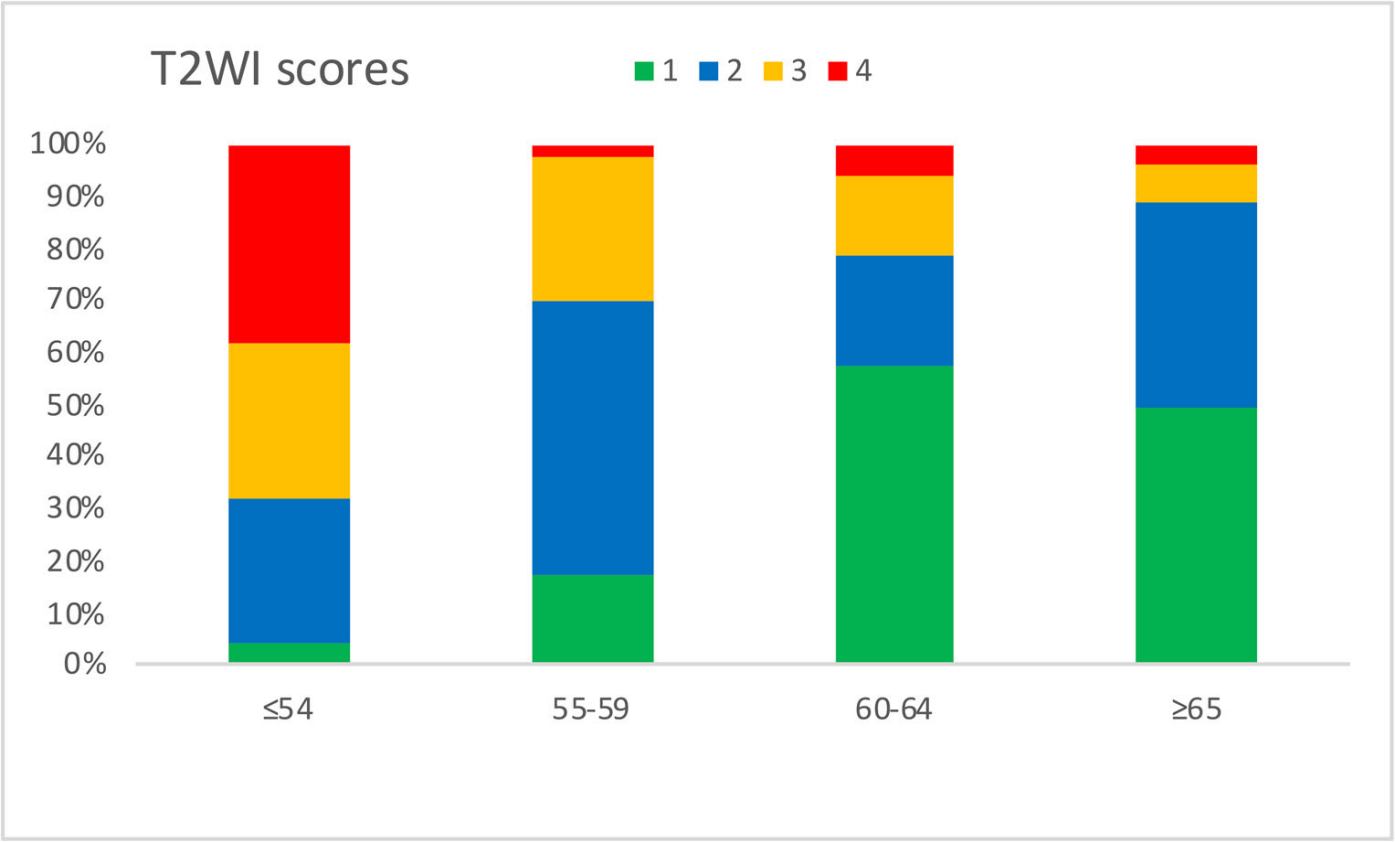
T2-weighted imaging (T2WI) score distribution among the four age groups (≤ 54, 55–59, 60–64, and ≥ 65 years); scores reduce with increasing age

The mean T2WI scores reduced with increasing age: 3.0 (patients ≤ 54 years), 2.2 (55–59 years), 1.7 (60–64 years), and 1.7 (patients ≥ 65 years) (Table 3 and Fig. 4). A similar inverse relationship was also seen with DWI and DCE scores (Table 3 and Supplemental figures 1 and 2). Cramér’s V coefficient showed a strong negative association between age groups and the three scores individually (*V* = 0.36 for age versus T2WI, *V* = 0.33 for age versus DWI, and *V* = 0.31 for age versus DCE). For the age group ≤ 54 years, scores 3–4 were seen in a higher proportion of patients at 68.1% for T2WI, 53.2% DWI, and 69.6% for DCE compared to patients aged ≥ 65 years at 10.9% for T2WI, 7.3% for DWI, and 25.0% for DCE. There was moderate agreement between the two readers for all scores, with the highest agreement demonstrated for T2WI, with a weighted *κ* = 0.58, followed by DCE with weighted *κ* = 0.47 and DWI score with weighted *κ* = 0.43.

**Table 3:** Mean background scores (T2WI, DWI, and DCE) for the different age categories

Age range	Mean T2WI score	Mean DWI score	Mean DCE score
≤ 54	3.02	2.70	3.13
55–59	2.15	1.70	2.40
60–64	1.70	1.45	1.91
≥ 65	1.65	1.42	1.88

*T2WI* T2-weighted imaging, *DWI* diffusion-weighted imaging, *DCE* dynamic contrast-enhanced imaging

**Fig. 5:**
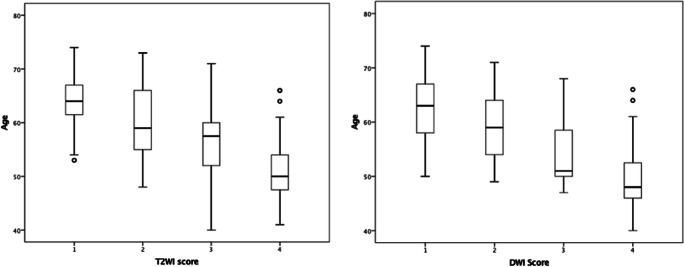
Relationship between age and T2-weighted imaging (T2WI) and diffusion-weighted imaging (DWI) scores. Scores reduce as age increases

There was a moderate, negative correlation between age and scores for each of the three multiparametric sequences (T2WI: *r* = − 0.52, DWI: *r* = − 0.49, DCE: *r* = − 0.45, all with *p* < 0.001), with higher scores associated with younger patients (Fig. 5 and Supplemental figure 3). Statistically significant positive correlations were also shown between the T2WI score and the other mpMRI sequence scores, DWI (*r* = 0.84, *p* < 0.001) and DCE, respectively (*r* = 0.80, *p* < 0.001).

### Normalized T2WI signal intensity and ADC values

**Fig. 6:**
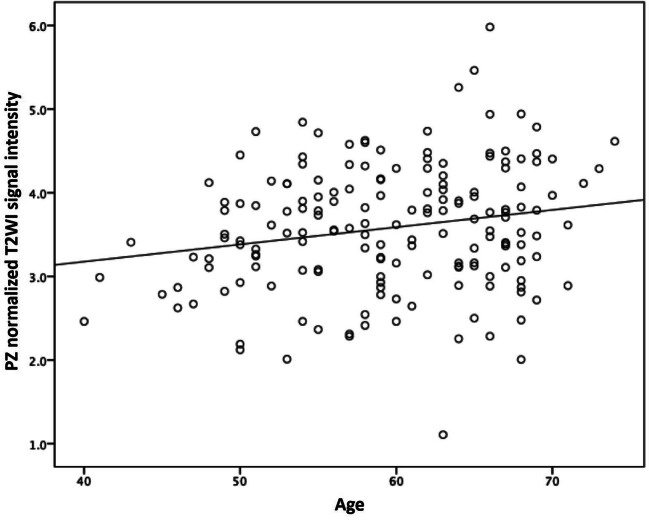
Relationship between age and peripheral zone (PZ) normalized T2-weighted imaging (T2WI) signal intensity. The graph shows T2WI signal intensity increases with increasing age

Normalized T2WI signal intensity and ADC values of the PZ were both lower for younger (≤ 54 years) than for the older age group (≥ 65 years), with mean normalized T2WI signal intensity values at 3.4 ± 0.67 versus 3.7 ± 0.79 and ADC values at 1.30 ± 0.20 versus 1.44 ± 0.18, respectively. A statistically significant, positive correlation was found between age and normalized PZ-T2WI signal intensity (*r* = 0.20, *p* = 0.009), shown in Fig. 6. There was also a statistically significant, positive correlation between age and mean PZ-ADC values (*r* = 0.33, *p* = 0.001; Supplemental figure 4).

## Discussion

The results of our study highlight a significant inverse relationship between age and background heterogeneity of the normal peripheral zone (PZ) on T2-weighted imaging (T2WI), diffusion-weighted imaging (DWI), and dynamic contrast-enhanced imaging (DCE) sequences. Younger patients showed significantly higher heterogeneity on background PZ scoring, and quantitative normalized T2WI signal intensity and apparent diffusion coefficient (ADC) values were reduced compared to those of older age.

To our knowledge, there are no previous studies mapping age-related background change in a biopsy-naïve population. Among other methodological benefits, this eliminates the potential confounder of intermediate-to-low T2WI signal intensity PZ changes relating to biopsy-related hemorrhage. There is limited data on the normal age-related appearances of the PZ at MRI. In concordance with our study, Allen et al in 1989 reported increased signal intensity of the PZ in older men on T2WI; however, the study was performed without DWI or DCE and did not include pathological confirmation [18]. More recently, De Visschere et al correlated PZ changes on MRI with histological features at prostatectomy and concluded that hyperintense T2WI signal intensity within the PZ reflects either simple or cystic atrophy, which predominantly occurred in older men [19]. Previous work is also supportive of the positive correlation between age and ADC values [23, 24]. Physiologically our findings can be explained by age-related alterations in sex hormones, with associated increases in luminal volume of prostatic acini and reduced glandular epithelium with increasing age [25, 26]. This results in a lower number of glands and a relatively higher water content in older men, and would be expected to be reflected in reduced restriction of diffusion and a higher T2WI signal intensity [19, 23]. Another interesting observation is the age at which changes in prostate volume occur, with the most marked differences noted between the groups < 54 years and 55–59 years old. This is consistent with earlier work showing prostatic growth rate peaks at 4.15 ± 4.98 cc/year in men aged 56–65 [16]. As expected, the overall gland increase is driven by TZ growth, with no significant changes in PZ volumes [27, 28]. Of note, our results suggest the background heterogeneity of PZ on all three sequences followed a similar age-related pattern with a stepwise change in the mid-50s, but a less notable decline afterwards, which is in concordance with the study by Shi et al who described increasing ADC values with age [24]. Not all men will undergo changes to the same extent and/or at this age cut-off, and other benign conditions such as inflammation, adenosis, and post-atrophic hyperplasia can all affect T2WI signal intensity, induce lower ADC values, and result in increased enhancement on DCE [19].

These results are of clinical importance and need to be appreciated when evaluating for potential clinically significant PCa in younger men, where background changes may make lesions less conspicuous on all mpMRI sequences. Two recent studies in men undergoing prebiopsy prostate MRI highlight this potential issue, with systematic biopsy showing a higher detection rate over targeted biopsy in men < 50 years [29] and mpMRI having a significantly lower sensitivity for clinically significant PCa in patients < 50 years old (49%) compared to men aged over 55 (73%) [30]. Beyond lesion detection, the ability to accurately stage the gland may also be compromised, as lesion-capsular contact is a key criterion, and may be more challenging to estimate in the context of diffuse background change [31–34]. The scoring system we propose may thus be a valuable addition to everyday clinical practice. The current PIRADS scoring system does not incorporate an assessment of the background gland; however, such an approach has been widely adopted in breast radiology by assigning a “Breast Composition Category (A–D)” to describe the breast density, which is known to correlate with the sensitivity of mammography interpretation [20]. The advantage of such a system is the clarity and meaning associated with higher density (or in our case heterogeneity) scores, which convey a message to clinicians that both sensitivity and specificity may be reduced. In such prostate patients, clinical parameters would likely play a more important role in biopsy decision-making and further tools such as risk calculators may be warranted [29].

Our study had several limitations. The scoring system assessed was based on a single-center experience and external validation, and multireader studies would be helpful to assess the generalizability of our results. Any biopsy technique is prone to sampling error, and it is therefore not possible to fully exclude the presence of undiagnosed low-volume PCa for all patients within the cohort. However, in the absence of prostatectomy for presumed benign disease, a negative systematic 12-core biopsy and negative clinical follow-up allow for a reasonable clinical standard. For cases with no biopsy, clinical follow-up was for a mean of 24.2 months and patients had a mean PSA density of 0.08 ng/mL^2^, providing reassurance of benignity. We excluded cases with clinically diagnosed prostatitis or pathology-reported acute or chronic prostatitis. Whilst local pathologists report to ISUP standards which require the presence of inflammatory lesions to be reported [35], it has been acknowledged that mild inflammatory changes are not always apparent on biopsy and/or may not be consistently reported by pathologists [36]. The prevalence of mild inflammatory change in patients with negative biopsy results is estimated to be 10% [37] and thus may have been under-represented in our cohort, making this a potential confounder; however, we would expect an even distribution of such cases across the cohort. We did not assess the TZ for background changes; tumors occur less frequently in this zone, and features of age-related benign prostatic hyperplasia have been well described previously [38]. Indeed, the “normal” TZ is already considered heterogeneous and is described as representing “organized chaos” in PIRADS; however, age-related background MRI assessment of the normal TZ could represent the basis for future work. DCE imaging was only assessed visually and without assessment of enhancement curve types; however, this is no longer required for post-contrast assessment according to current PIRADS guidelines [10]. Finally, we did not assess the scoring system in relation to cancer detection. This could be the focus of future work, wherein background PZ scores could be correlated prospectively with lesion conspicuity, and PIRADS scores and the detection of clinically significant PCa, particularly compared to background systematic biopsy cores.

In conclusion, our results show a significant relationship between age and the normal background signal intensity of the prostate peripheral zone, with younger men exhibiting lower T2-weighted imaging (T2WI) signal intensity, lower apparent diffusion coefficient (ADC) values, and diffuse enhancement on dynamic contrast-enhanced imaging (DCE), which may hinder interpretation. We propose a standardized scoring system for evaluating this background change, which may help convey the potential for diagnostic uncertainty to clinicians.

## Supplementary Information

ESM 1 (DOCX 141 kb)

## Abbreviations

ADC: Apparent diffusion coefficient
BIRADS: Breast Imaging Reporting and Data System
DCE: Dynamic contrast-enhanced imaging
DWI: Diffusion-weighted imaging
mp: Multiparametric
PCa: Prostate cancer
PIRADS: Prostate Imaging Reporting and Data System
PZ: Peripheral zone
T2WI: T2-weighted imaging
TZ: Transition zone
WG: Whole gland
