# Supplementary material for: MRI features of the normal prostatic peripheral zone: the relationship between age and signal heterogeneity on T2WI, DWI, and DCE sequences
Source: Eur Radiol. 2021 Jan 4;31(7):4908–17. doi: 10.1007/s00330-020-07545-7 (PMC8213603; doi:10.1007/s00330-020-07545-7)
Supplement: Supplementary file 1 — (DOCX 141 kb) [file 330_2020_7545_MOESM1_ESM.docx]

**ELECTRONIC SUPPLEMENTARY MATERIAL**

**Supplemental table 1. MRI protocol at 1.5T and 3T**

| **Parameter** | **Axial T2 FSE** | | **Axial DWI** | | **DCE LAVA** | |
| --- | --- | --- | --- | --- | --- | --- |
|  | **1.5T** | **3T** | **1.5T** | **3T** | **1.5T** | **3T** |
| **TR/TE (ms)** | 5048/73 | 3743/102 | 3400/62 | 3775/85 | 6.2/3.1 | 4.3/1.8 |
| **FOV (cm)** | 240x240 | 180 | 240x240 | 280 | 240x240 | 240 |
| **Acquisition matrix** | 352x352 | 384x384 | 128x128 | 128x128 | 256x256 | 256x256 |
| **Slice thickness (mm)** | 3.5 | 3 | 4 | 3 | 4 | 3 |
| **Gap (mm)** | 0.5 | 0 | 0 | 0 | 0 | 0 |
| **b-values (s/mm^2^)** | - | - | 100, 550, 1000, 1400 | 100, 750, 1400, 2000 | - | - |
| **Synthetic b-values (s/mm^2^)** | - | - | 1400, 2000 | 2000, 2500 | - | - |
| **Echo Train Length** | 16 | 16 | Single shot | Single shot | 1 | 1 |
| **Temporal resolution** | - | - | - | - | 10s | 7s |


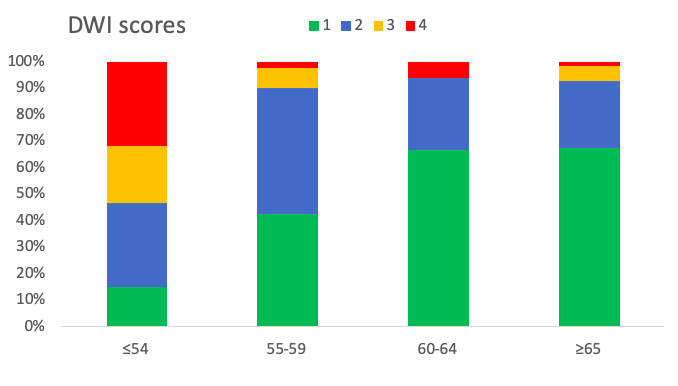


**Supplemental figure 1.** Diffusion-weighted imaging (DWI) scores distribution among the four age groups (≤54, 55-59, 60-64 and ≥65 years); scores reduce with increasing age.

**
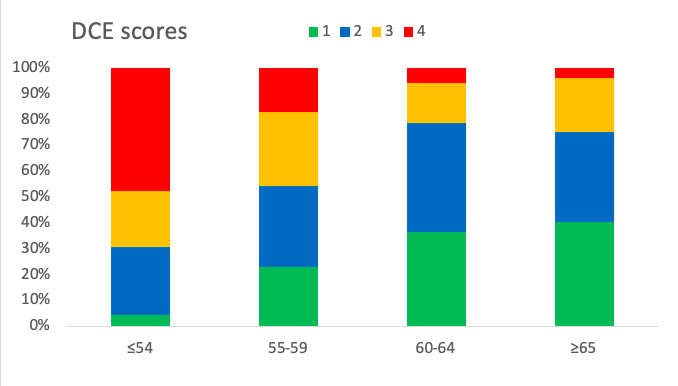
**

**Supplemental figure 2.** Dynamic contrast-enhanced imaging (DCE) scores distribution among the four age groups (≤54, 55-59, 60-64 and ≥65 years); scores reduce with increasing age.

**
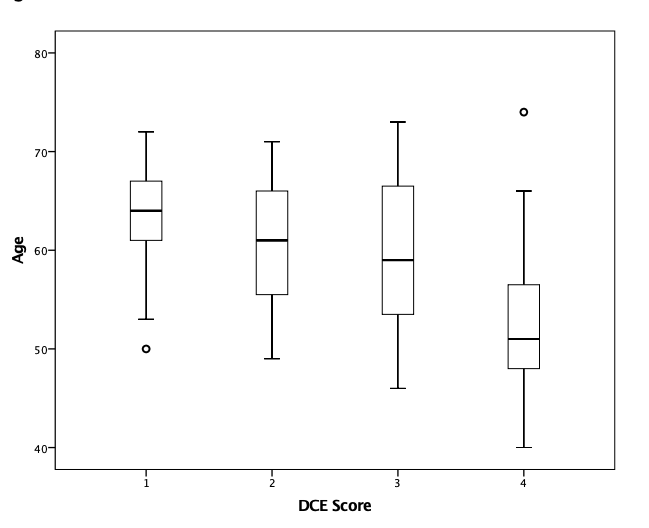
**

**Supplemental figure 3.** Relationship between age and dynamic contrast-enhanced (DCE) scores. Scores reduce as age increases.

**
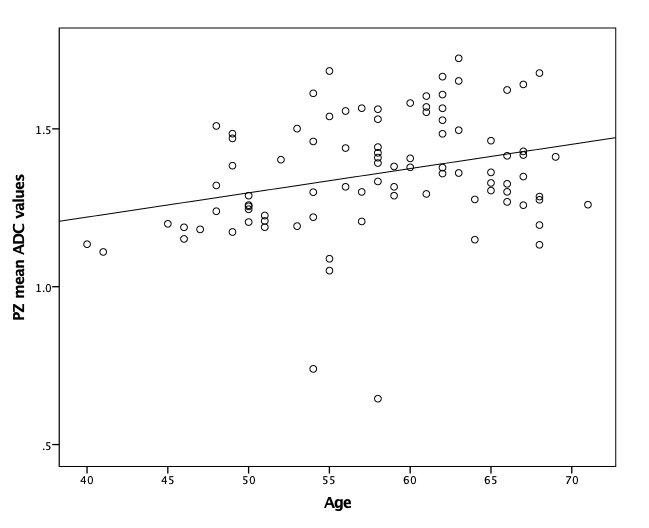
**

**Supplemental figure 4.** Relationship between age and mean apparent diffusion coefficient (ADC) values of peripheral zone (PZ). The graph shows ADC values increase with increasing age.
